# Fabrication of a counter electrode for dye-sensitized solar cells (DSSCs) using a carbon material produced with the organic ligand 2-methyl-8-hydroxyquinolinol (Mq)

**DOI:** 10.1039/c9na00206e

**Published:** 2019-06-27

**Authors:** Rahul Kumar, Veena Sahajwalla, Parag Bhargava

**Affiliations:** Department of Metallurgical Engineering and Materials Science, Indian Institute of Technology Bombay Mumbai India 400076 kumarrahul003@gmail.com; Centre for Sustainable Materials Research and Technology, School of Materials Science and Engineering, University of New South Wales Sydney NSW 2052 Australia

## Abstract

Dye sensitized solar cells (DSSCs) are low cost solar cells and their fabrication process is easy relative to silicon based solar cells. Platinum can be replaced with carbon materials as counter electrodes in DSSCs because of their good catalytic properties and low cost. A carbon material was produced by carbonization of an organic ligand (2 methyl 8-hydroxy quinolinol (Mq)) at high temperature in flowing argon gas. Polyvinylpyrrolidone (PVP) was used as a surfactant for making carbon slurry from carbon produced using Mq. For the fabrication of the counter electrode, a carbon coating was prepared by using the doctor blading technique and the carbon slurry was coated on the FTO substrate. DSSCs based on the carbon counter electrode exhibit a higher *V*_oc_ of 0.75 V than that of the Pt counter electrode (0.69 V). DSSCs based on the carbon material showed a power conversion efficiency (PCE) of 4.25% and fill factor (FF) of 0.51 which are slightly lower than those of the platinum (Pt) based counter electrode which showed a PCE of 5.86% and FF of 0.68.

## Abstract 2

DSSCs fabricated using a carbon material showed a *J*_sc_, *V*_oc_, and *η* of 11.00 mA cm^−2^, 0.75 V, and 4.25% respectively.

## Graphical abstract

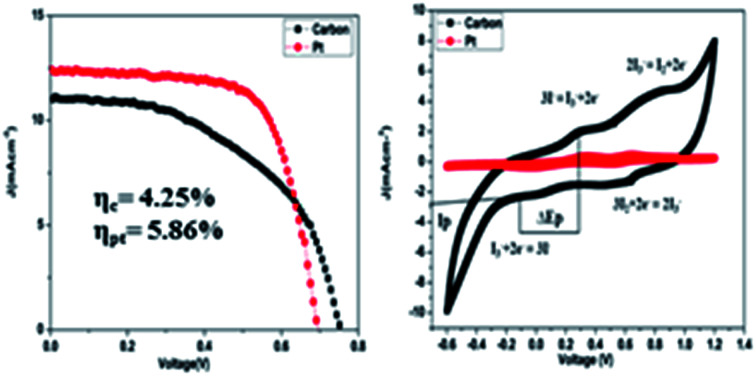

## Introduction

Energy is an important need for the growth of human civilization. Currently, the world's energy demand is basically accomplished using fossil fuels (coal, petroleum and natural gases). Fossil fuels have the main problem that they are non-renewable and generate CO_2_ and other greenhouse gases. There are other renewable energy sources like wind and hydropower, but they have high organization and maintenance costs.^1–7^ Solar energy is a continuous and renewable source of energy. At present, silicon based solar cells are considered the main commercialized technology by which we generate electricity, but the fabrication cost of silicon solar cells is high.^8^ Dye sensitized solar cells (DSSCs) have attracted much attention because of their low cost, environmental friendliness, high energy conversion efficiency, and simple fabrication procedure.^9–17^ A DSSC is constituted of a counter electrode which is generally made of Pt, a photoanode which is sensitized with a dye, and an electrolyte containing a redox couple (I_3_^−^/I^−^). Platinum is a costly, low abundance noble metal and it suffers from slow dissolution because of the corrosive redox electrolyte, which decays its long-term stability. These are the drawbacks that prevent the large-scale application of the Pt electrode in DSSCs.^18–20^ At present, a vital problem is a search for economical and highly catalytic materials to supersede Pt for DSSC development.^21–24^ Some other low cost materials, such as carbon materials,^25–34^ alloys,^35^ conductive polymers,^36–39^ metal carbides,^40^ metal oxides,^41,42^ and transition metal based materials including metal nitrides,^43–46^ metal sulphides and metal alloys,^47–49^ have been utilized as counter electrodes and shown very good catalytic activity for the triiodide reduction. Carbon materials are very attractive materials to supersede the platinum electrode in DSSCs because of their high electronic conductivity, large surface area, low cost, environmental friendliness, corrosion resistance towards iodine, high reactivity for triiodide reduction, and availability. Several carbon materials such as graphite, graphene, carbon black, glassy carbon, carbon nanotubes, activated carbon, nanocarbon, carbon fibers, well-ordered mesoporous carbon, carbon produced from discarded toners of printer, carbon quantum dot, fullerene, conductive carbon paste, carbon derived from human hair, graphene composites, N and P doped graphene, 3D SWCNTs/graphene aerogel carbon derived from saccharide and sugar-free, carbon derived from organic ligands, carbon derived from Triton-X, *etc.* have been successfully employed as counter electrodes.^25–34^ Organic compounds, which have carbon (C) hydrogen (H), and nitrogen (N) atoms, are converted into graphitized carbons after carbonization at high temperature.^34^ Nitrogen allows one to obtain strong and thermally stable carbon materials and may participate in the final formation of oriented graphite compounds.^50^ It is well known that carbons derived from N containing compounds normally have abundant porosity, tunable surface properties and high stability.^51^ In this work, the preparation of the counter electrode was done by using a carbon material produced by carbonization of an organic ligand (2-methyl-8-hydroxy quinolinol (Mq)) at 1200 °C in flowing argon gas. It was expected that from the carbonization of the organic ligand, a good quality carbon material would be obtained.

## Experimental

### Materials

An organic ligand (2-methyl-8-hydroxy quinolinol, Sigma Aldrich 98%, chemical formula C_10_H_9_NO) was used as the carbon material. Fluorine doped tin oxide (FTO) (TEC8, sheet resistance 8–9 Ω □^−1^, Pilkington) was used as the substrate to prepare the photoanodes and counter electrodes. Polyvinylpyrrolidone (PVP), (Sigma Aldrich, K-32) was used as the surfactant and ethanol (Changshu Yangyuan Chemical, China AR Grade) was used as the solvent for the preparation of the carbon-based counter electrodes. TiO_2_ nanopowder (P25, Degussa) was used as the photocatalyst, while polyethylene glycol (*M*_w_ = 600) PEG-600 (Thomas Baker) was used as the dispersant to prepare the photoanodes. RuL_2_(NCS)_2_ (L = 2,2-bipyridyl-4,4-dicarboxylic acid) (known as N_3_ dye) (Dyesol) was used as the photosensitizer for the fabrication of the cells. For the preparation of the electrolyte, guanidinium thiocyanate (0.10 M), 4-*tert*-butylpyridine (0.50 M) [TBP] (96% Aldrich), 1-methyl-3-propylimidazolium iodide [PMII] (≥98% Aldrich) (0.60 M) and iodine (LR, Thomas Baker) [I_2_] (0.03 M) were used.^31–34^

### Synthesis of the carbon material and fabrication of the counter electrode

For the fabrication of the counter electrode for DSSCs, the carbon material was obtained by carbonization of the organic ligand (2-methyl-8-hydroxy quinolinol (Mq)) at 1200 °C in flowing argon gas. The carbon material appeared flaky in nature. To make the carbon material a finer powder it was crushed in a mortar and pestle. For the preparation of carbon slurry, initially, PVP was added to ethanol and kept on a stirrer for stirring for half an hour to make a homogeneous solution. After making the PVP solution, the carbon powder was mixed with the PVP solution and the carbon mixture was kept on a pot mill for 48 hours to reduce the deagglomeration of the carbon powder and make a homogeneous slurry. The PVP to carbon weight ratio was 1 : 8. Counter electrodes were prepared on an FTO substrate by coating the carbon slurry using the doctor blade technique. Scotch tape (3M) was used to coat the carbon slurry on the required area. After this, the carbon counter electrodes were dried in an oven at 50 °C and then placed in a furnace at 450 °C for 1 hour in flowing argon gas in order to burn out the binder. A sputter deposited platinum counter electrode was also fabricated for comparison with the carbon counter electrode.^31–34^

### Fabrication of photoanodes and dye loading

To fabricate the photoanodes, titania slurry was fabricated by roller milling of TiO_2_ nanopowder, PEG 600 (dispersant) and ethanol (solvent) for 24 h. The prepared slurry has been used to fabricate the photoanodes. The prepared slurry was coated onto the cleaned FTO substrates and sintering was done at 450 °C for 1 h in flowing argon gas. After this, the electrodes were cooled to 80 °C and dipped into a 0.3 mM solution of N_3_ dye in ethanol. The electrodes were removed from the dye solution after 24 h and then rinsed with ethanol and dried. It is expected that the amount of dye adsorbed by the photoanodes for both the devices would be the same as the photoanodes were prepared at the same time and using the same procedure.^31–34^

### Preparation of electrolyte

The electrolyte used in the DSSCs was fabricated by mixing of guanidinium thiocyanate (0.10 M), iodine (LR, Thomas Baker) [I_2_] (0.03 M), 4-*tert*-butylpyridine (0.50 M) [TBP] (96% Aldrich), 1-methyl-3-propylimidazolium and iodide [PMII] (≥98% Aldrich) (0.60 M), in the mixture of valeronitrile and acetonitrile (volume ratio: 15 : 85). The solution was kept on a magnetic stirrer for stirring for around 30 minutes.^31–34^

### Fabrication of DSSCs

For the preparation of DSSCs, counter electrodes and photoanodes were prepared separately as explained above. DSSCs were prepared using the counter electrode, electrolyte, photoanode, and spacer. A 25 μm spacer was used to combine both the electrodes together. The spacer was kept on the photoanode and heated to bond with it. The electrolyte was filled in the gap defined by the spacer and the carbon or platinum coated FTO substrate (counter electrode) was kept over it. Binder clips were used to hold the assembly together. The active area of the DSSCs was 0.25 cm^2^.^31–34^

### Characterization

The carbon material was produced by carbonization of the organic ligand (2-methyl-8-hydroxy quinolinol (Mq)) at 1200 °C. The produced material was characterized by using different techniques such as, Brunauer–Emmett–Teller (BET) (Smart Sorb 92/93, Smart Instruments Co.), X-ray powder diffraction (XRD) (Expert Pro, 40 kV, 30 mA, Panalytical), Raman spectroscopy (Jobin-Yvon, France Ramnor HG-2S Spectrometer), field emission gun electron transmission microscopy (FEG-TEM) (JEOL, JEM-2100F) and field emission gun electron scanning microscopy (FEG-SEM) (JEOL, JSM-7600F) independently.

Cyclic voltammetry (CV) of both the counter electrodes (Pt and carbon-based counter electrode) was performed using a three-electrode assembly at a scan rate of 50 mV s^−1^ from −0.6 to +1.2 V where the platinum or carbon coated FTO-substrate, Ag/AgCl and platinum wire worked as the working electrode, reference electrode and counter electrode respectively. The electrolyte solution consisted of 0.5 mM iodine, 0.1 M lithium perchlorate and 5 mM LiI in acetonitrile.^31–34^

The electrochemical impedance spectra (EIS) of the DSSCs were recorded in a frequency range of 100 kHz to 0.1 Hz under AM 1.5G simulated solar illumination at 100 mW cm^−2^. The ac amplitude and applied bias voltage were fixed at open circuit voltage (*V*_oc_) at 5 mV and 0 mV respectively. The EIS spectra of DSSCs were recorded using Zsimpwin software using two different equivalent circuit models. The thickness of the platinum films and TiO_2_ film was measured using a profilometer (Dektak XT profilometer, Icon Analytical Equipment Pvt. Ltd.).

The photo–current–voltage (*I*–*V*) characteristics of the DSSCs were measured by using a Keithley model 2420 source measure unit. The irradiation source was a 150 W xenon lamp on a Newport solar simulator with AM 1.5G. The active area of the DSSCs was 0.25 cm^2^.^31–34^

## Results and discussion

**Fig. 1:**
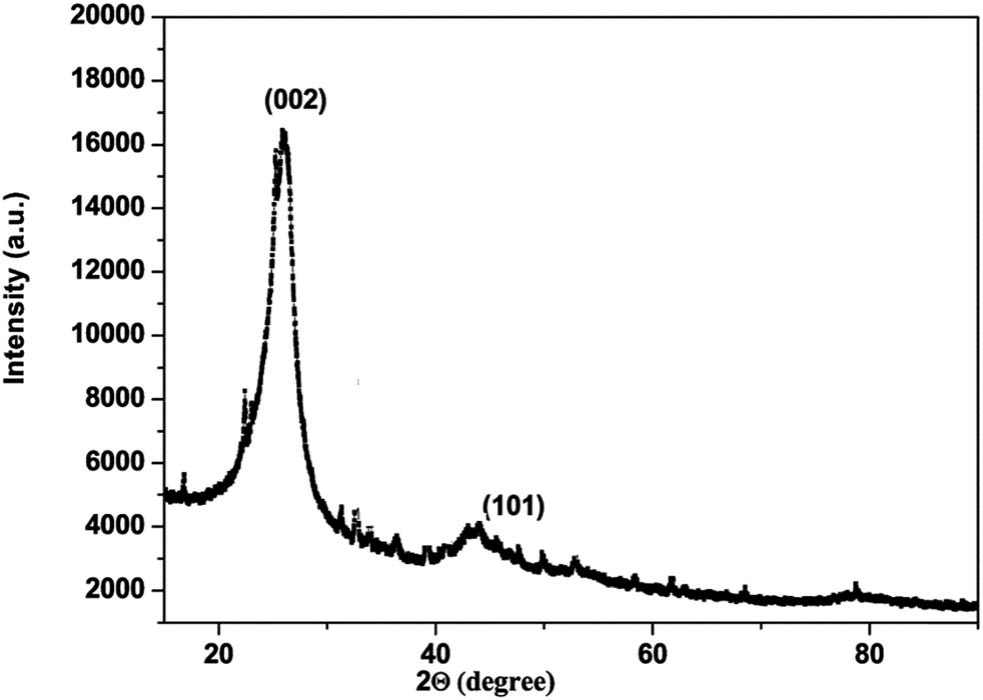
XRD pattern of carbon produced from Mq.

**Fig. 2:**
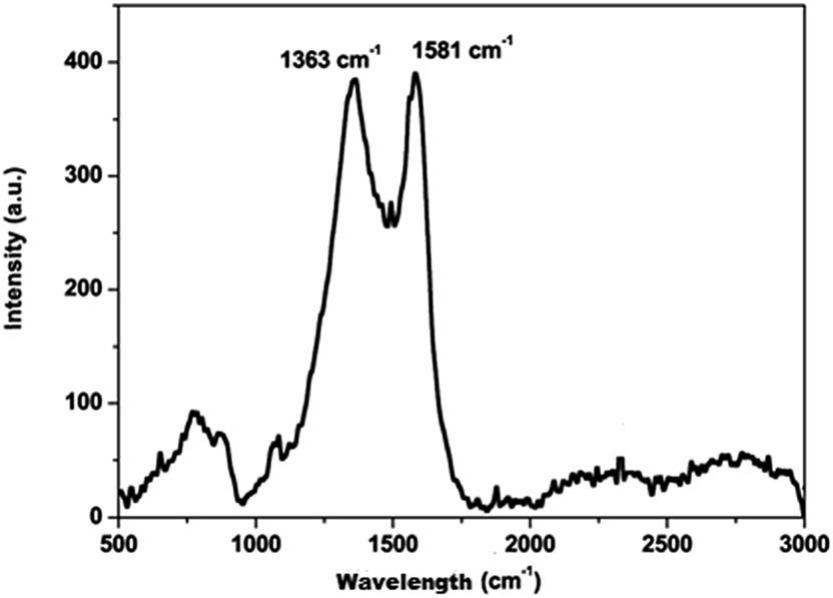
Raman spectra of carbon produced from Mq.

The carbon material produced using Mq was examined by using different techniques such as BET, XRD, Raman spectroscopy, FEG-TEM, FEG-SEM and cyclic voltammetry (CV). The carbon material produced using Mq was characterized by the BET method and the surface area of the carbon material was obtained as 17 m^2^ g^−1^. XRD measurement was done with a scan rate of 2 degree per min with a Cu-Kα source. The XRD pattern of the carbon material is shown in Fig. 1. Two peaks were obtained, which are commensurate with the crystalline reflections from the (101) and (002) planes respectively. The maximum intensity at which 2*θ* values are 43.63/26.40 was obtained for the (101)/(002) planes. The produced carbon material was characterized by Raman spectroscopy as shown in Fig. 2. Raman spectroscopy measurement was performed with a 514 nm excitation source with a scan rate of 2000 cm^−1^ min^−1^. Two prominent bands were found in Raman spectra. One band was found at 1363 cm^−1^, and this band is known as the D band and the D band is a feature of disorder such as defects, vacancies, bond disruption, *etc.* made in the carbon lattice. We have observed one another band at 1581 cm^−1^, and this band is known as the G band and the G band is a signature of the graphitic structure and is produced because of the in-plane vibrational modes of the sp^2^ bonded carbon atoms. Both the bands appear in the carbon-based materials.^52–58^ The *I*_D_/*I*_G_ ratio of the carbon material was found to be 0.98. The crystallite size (La) was measured by using the wavelength power law formula of the produced carbon material as given below using eqn (1).^59^

1 La (nm) = (2.4 × 10^−10^) *λ*_laser_^4^ (*I*_D_/*I*_G_)^−1^

where *λ* is the excitation laser wavelength in nm units, and *I*_D_/*I*_G_ is the ratio of the D and G band intensity.

**Fig. 3:**
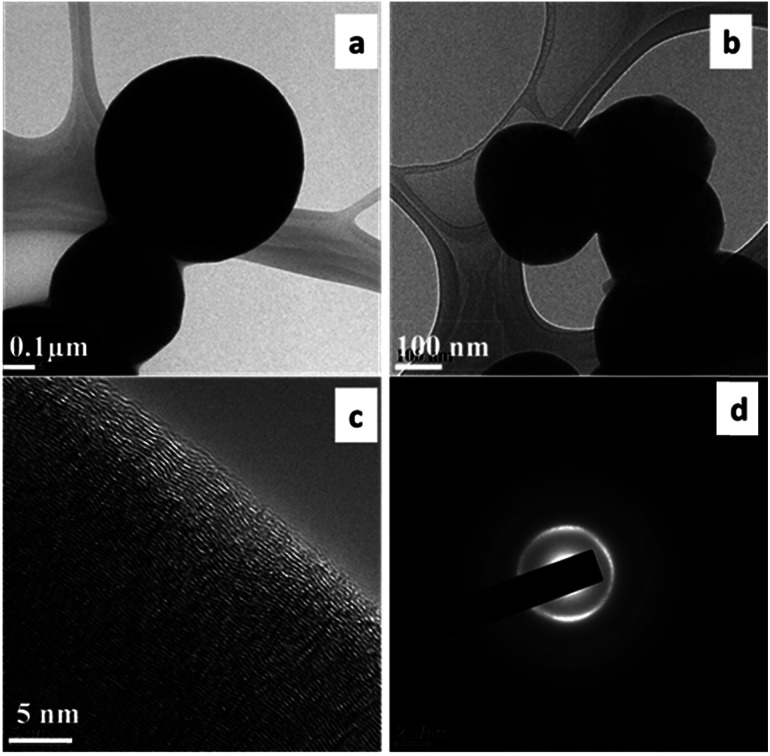
FEG-TEM micrographs (a & b) at low magnification and (c) at high magnification, and (d) the diffraction pattern of carbon produced from Mq.

The crystallite size of the carbon was measured to be 19.49 nm. FEG-TEM was utilized to characterize the morphology and nature of the carbon material. Fig. 3(a and b) demonstrate that the carbon particles are spherical in shape as shown in the FEG-TEM micrographs. Fig. 3(c) shows the crystalline nature of the carbon material. The diffraction pattern (Fig. 3(d)) of the carbon material indicates polycrystalline nature.

**Fig. 4:**
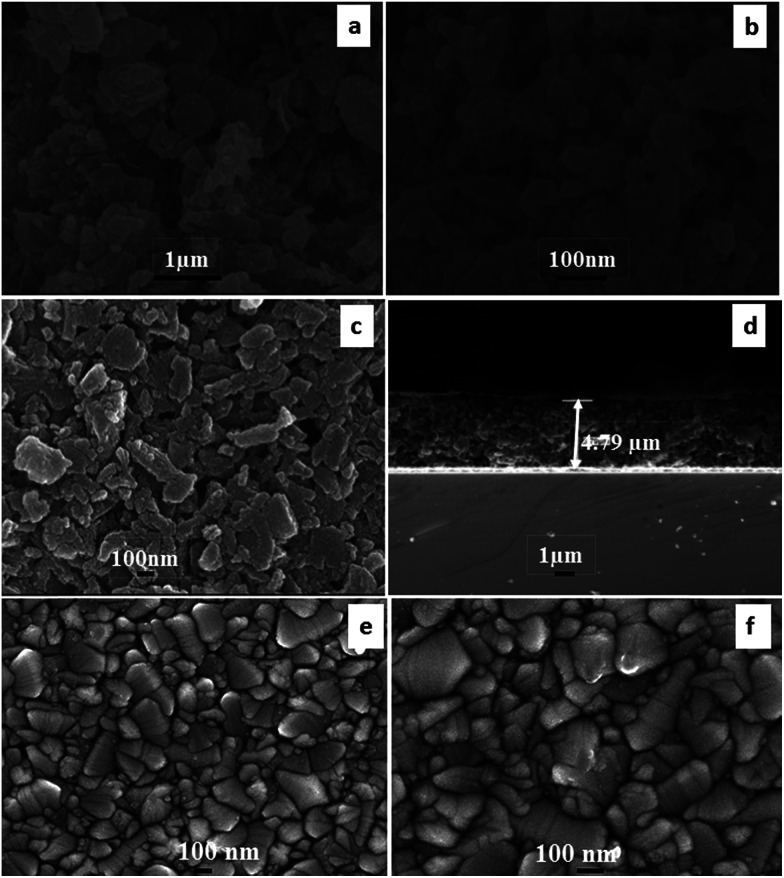
FEG-SEM micrographs (a) at low magnification and (b) at high magnification of carbon produced from Mq, (c) the upper surface of the carbon film, (d) side view of the carbon film sintered at 450 °C, and (e) & (f) the upper surface of the sputtered platinum film.

The surface morphology of the carbon material and counter electrode after heat treatment at 450 °C (1 hour) in flowing argon gas was characterized by FEG-SEM as demonstrated in Fig. 4. The morphology of the produced carbon material can be seen in (Fig. 4(a and b)). Carbon particles produced using Mq seem to be a combination of spherical and elliptical particles. It was expected that the surfactant (PVP) is transformed into carbon after heat treatment. The film seems to be porous in character and pores were present nonuniformly within the film as demonstrated in Fig. 4(c). The thickness of the film was examined by FEG-SEM as demonstrated in Fig. 4(d) and found to be 4.79 μm. The thickness of the film was chosen on the basis of our previous study, resulting in a high efficiency of DSSCs.^60^ The sputtered platinum film seems to be dense in nature as demonstrated in Fig. 4(e and f). The thickness of the platinum film was examined using a profilometer and it was found to be 40 nm.

**Fig. 5:**
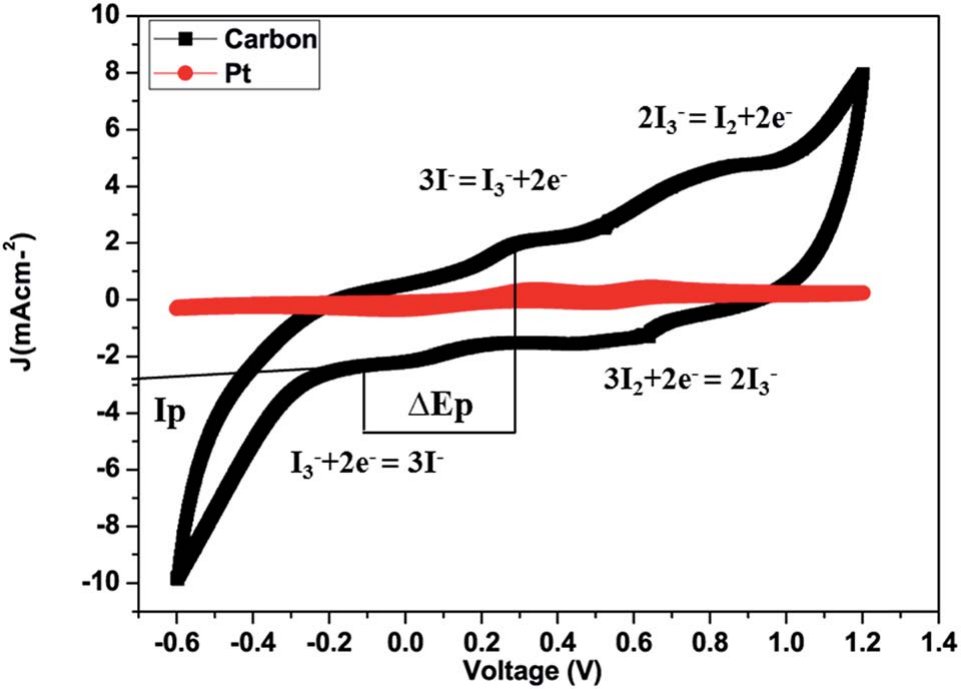
Cyclic voltammetry curves of the carbon counter electrode at a scan rate of 50 mV s^−1^ and Pt counter electrodes at a scan rate of 50 mV s^−1^ from −0.6 to +1.2 V in 5 mM LiI, 0.5 mM I_2_ and 0.1 M LiClO_4_ acetonitrile solution. Reference electrode: Ag/Ag^+^ in acetonitrile. Auxiliary electrode: Pt/FTO.

The cyclic voltammograms (CV) of platinum and carbon electrodes are shown in Fig. 5. The CV of the counter electrodes was carried out using a three-electrode assembly at a scan rate of 50 mV s^−1^ from −0.6 to +1.2 V where the platinum or carbon-coated FTO substrate, Ag/AgCl, and platinum wire, worked as the working electrode, reference electrode and counter electrode respectively. The electrolyte solution was prepared from 5 mM LiI, 0.5 mM iodine, and 0.1 M lithium perchlorate in acetonitrile. Fig. 5 shows the CV of the I_2_/I^−^ system for carbon and platinum electrodes. In CV curves we obtained two pairs of redox peaks and the peaks obtained on the positive side are considered as anodic peaks and the negative peaks are considered as cathodic peaks. In the CV curves, the anodic peaks correspond to the oxidation of iodide and triiodide and the cathodic peaks refer to the reduction of triiodide.

The relative negative pair is defined by redox reaction (2) and the positive one is defined by redox reaction (3).

2 I_3_^−^ + 2e^−^ = 3I^−^

3 3I_2_ + 2e^−^ = 2I_3_^−^

The carbon material produced using Mq exhibited both oxidation and reduction peaks which are comparable with those of the Pt electrode. The same behaviour of the carbon material signifies that the catalytic behavior of the carbon material is comparable to that of Pt. The carbon film made using carbon slurry has a porous structure made using the doctor blade technique and each carbon particle has catalytic sites which facilitate I_3_^−^ reduction. The redox reaction reaction (eqn 2) which occurs at the counter electrode of a DSSC and examine the catalytic capability and performance of the counter electrode. The redox reaction (eqn 3) which occurs in CV because of the reduction of I_2_ molecules.^61,62^

The peak current density (*I*_p_) and peak-to-peak separation (Δ*E*_p_) are two important parameters for estimating the catalytic activities of the electrodes. A high *I*_p_ value represents the high catalytic activity of the CE and a low Δ*E*_p_ represents a smooth redox reaction on the CE surface because Δ*E*_p_ is inversely proportional to the charge transfer rate; thus, the catalytic activity is enhanced with decreasing Δ*E*_p_.^63–66^ It was observed that the Ip value of the redox peak for the carbon produced from Mq (*I*_p_ = −2.90 mA cm^−2^) is higher than that of the Pt CE (*I*_p_ = −0.40 mA cm^−2^), and the Δ*E*_p_ value of the carbon produced from Mq (Δ*E*_p_ = 0.31 V) is lower than that of the Pt CE (Δ*E*_p_ = 0.37 V) as demonstrated in Fig. 5. Both the values (Δ*E*_p_ and *I*_p_) for the carbon produced from Mq indicate that the carbon material shows high redox activity towards I_3_^−^ and I_2_ even higher than that of Pt.^67^ The comparison of Δ*E*_p_ and *I*_p_ values for various carbon-based materials is given in Table 1.

**Table 1:** Δ*E*_p_ and *I*_p_ values for various carbon-based materials^a^

Carbon-based material	Δ*E*_p_ (V)	*I*_p_ (mA cm^−2^)	Reference
A-CNT	0.34	−0.95	68
F-MC	0.42	−2.82	69
O-NCI	0.57	−1.70	70
MWCNTs	0.77	—	71
CB	0.64	—	72
APNT	0.49	—	73
N-CCS	0.35	—	74
BCM-PW	0.58	−1.08	75
BCM-FT	0.53	−1.57	75
BCM-FP	0.54	−1.49	75
BCM-PL	0.58	−1.19	75
SDC	0.70	—	60
Mq	0.31	−2.90	Present work

a Activated carbon nanotubes (A-CNT), functionalized mesoporous carbon (F-MC), organic nanocarbon ink (O-NCI), multiwalled carbon nanotubes (MWCNTs), carbon black (CB), activated polypyrrole nanotubes (APNT), N-doped core–shell carbon spheres (N-CCS), biowaste derived carbon materials (BCM) (PW: Phoenix wood, FT: facial tissue, FP: filter paper, PL: palm leaf), and sucrose derived carbon (SDC).

**Fig. 6:**
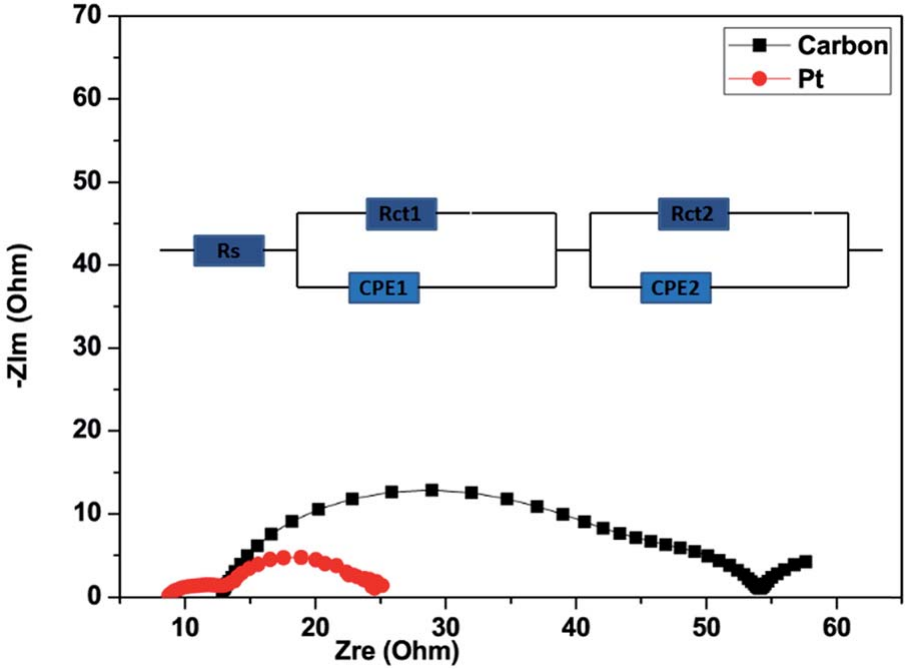
Nyquist plots of electrochemical impedance spectra of DSSCs made using carbon produced from Mq and Pt measured at open circuit voltage (*V*_oc_) from 100 kHz to 0.1 Hz under 1 sun illumination (AM 1.5, 100 mW cm^−2^).

To investigate the catalytic activity of the carbon material and Pt counter electrodes (CEs), we carried out electrochemical impedance spectra (EIS) measurements. Nyquist plots for the carbon and Pt are shown in Fig. 6. As can be observed from Fig. 6, the Nyquist plots have two dominant semicircles.^76^ The circuit model, which was used to fit the experimental data is shown in the inset of Fig. 6. The first semicircle, which can be seen in the high-frequency region (100–0.1 kHz), represents the impedance of charge-transfer (*R*_ct_1__) at the counter electrode and the first semicircle also represents the sheet resistance (*R*_s_), which is governed by the FTO/glass substrate and the thickness of CEs. The second semicircle, which can be seen in the middle-frequency range (100–1 Hz), is mainly contributed by the charge-transfer (*R*_ct_2__) process at the TiO_2_/dye/electrolyte interface.^77^ The constant phase element (CPE) is used to model the capacitance that originates at the electrode/electrolyte interface because of the accumulation of ions at the electrode surface. *R*_s_, *R*_ct_1__, and *R*_ct_2__ represent the fitted values of the equivalent circuit for EIS inserted in Fig. 6 and are listed in Table 2. The sum of *R*_s_, *R*_ct_1__, and *R*_ct_2__ is the total internal series resistance (*R*_total_).^78^ The total internal series resistance for DSSCs fabricated using carbon and Pt is 54.5 Ω and 24.97 Ω respectively. It can be observed that the Pt CE exhibits better performance. In both the experiments, the photoanode electrodes are the same. It was observed that *R*_ct_1__ is affected by the counter electrodes, so the fitting value *R*_ct_1__ of the carbon-based CE is higher than that of Pt. The smaller *R*_ct_1__ (7.07 Ω) of the Pt CE as compared to that of the carbon CE (17.3 Ω) suggests that Pt has more capability for electrocatalytic reduction of I_3_^−^ to I^−^ ions in an electrolyte as compared to the carbon CE.

**Table 2:** The fitted EIS parameters of DSSCs with different counter electrodes fabricated using carbon and Pt

Device	*R*_s_ (Ω)	*R*_ct_1__ (Ω)	*R*_ct_2__ (Ω)	*R*_total_ (Ω)
Carbon	13.9	17.3	23.3	54.5
Platinum	9.2	7.07	8.7	24.97

**Fig. 7:**
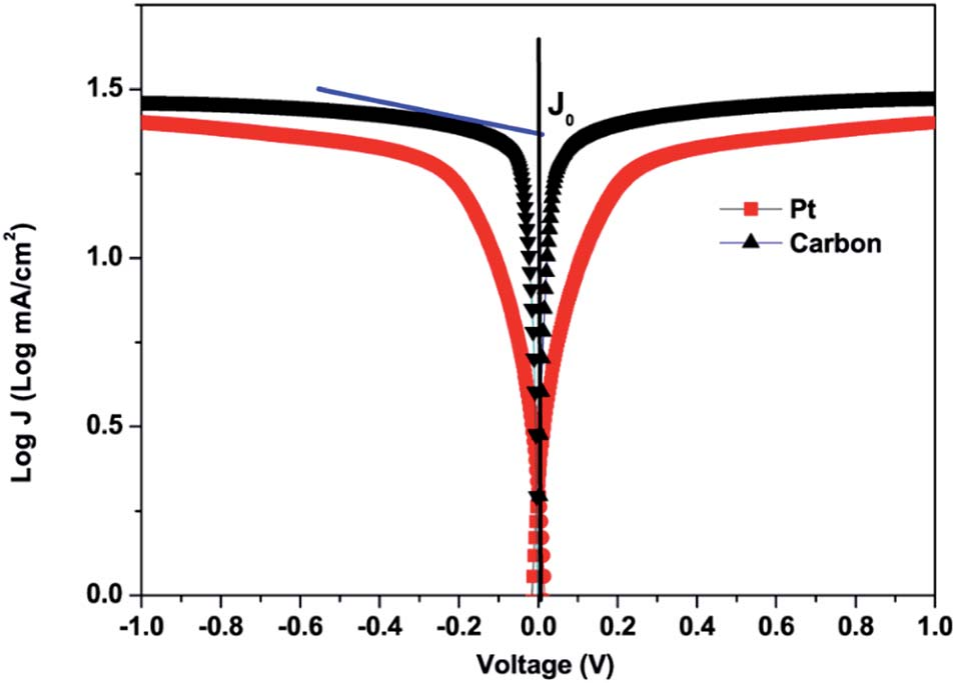
Tafel polarization curves of carbon and Pt counter electrodes obtained for symmetrical cells.

The Tafel-polarization test was done to further study the electrocatalytic activities of platinum and carbon CEs. Fig. 7 shows the Tafel curves of the carbon and platinum CEs, which were derived from symmetrical cells. These cells were prepared with two identical electrodes and were separated by a 60 μm thick Surlyn film. The symmetrical cells were prepared with the same electrolyte, which was used to fabricate DSSCs. The exchange current density (*J*_0_) of the electron transfer kinetics at the electrolyte/CE interface under equilibrium conditions can be directly observed from the Tafel polarization curve. It can be obtained when the overpotential is zero from the intercept of the extrapolated linear region of the anodic or cathodic branch.^54^ The equation between *J*_0_ and *R*_ct_ can be written as demonstrated in eqn (4):

4 *J*_0_ = *RT*/*nFR*_ct_

where *J*_0_ is the exchange current density on the electrode surface, *R* is the gas constant, *T* is the absolute temperature, *R*_ct_ is the charge-transfer resistance, *F* is Faraday's constant and *n* is the number of electrons involved in the reduction of I_3_^−^.^79–81^ A better catalytic activity is observed for a higher *J*_0_ value. It can be observed that carbon CEs show a higher value than platinum-based CEs. This implies that carbon CEs exhibit better catalytic activity. The CEs prepared using the carbon material show a higher *J*_0_ value, and this may be due to a result of the higher active surface area of the carbon material. It is expected that CEs prepared using the carbon material produced from Mq will exhibit higher stability than the Pt CEs. It is already reported by many research groups that carbon-based materials are more stable than Pt as CEs in DSSC applications.^82–85^

**Fig. 8:**
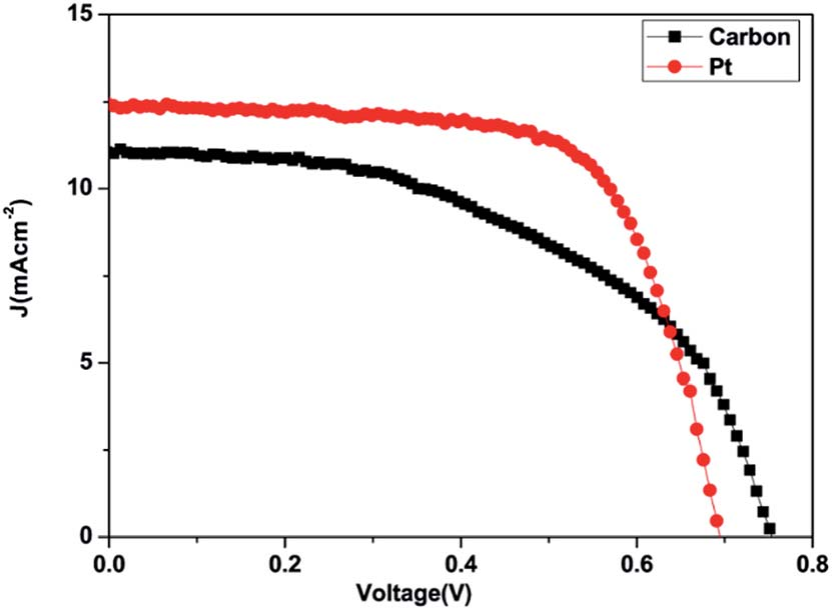
Photocurrent–voltage characteristics of the cells made using the carbon and Pt counter electrodes with N_3_ dye and TiO_2_ paste.

The *J*–*V* characteristic curves of DSSCs using the carbon material and Pt counter electrodes are shown in Fig. 8. The photovoltaic performance parameters of the DSSCs are given in Table 3.

**Table 3:** Photovoltaic parameters of DSSCs with different counter electrodes fabricated using the carbon and Pt

Counter electrode	*J*_sc_ (mA cm^−2^)	*V*_oc_ (V)	FF	*η* (%)
Platinum	12.40	0.69	0.68	5.86
Carbon derived from Mq	11.00	0.75	0.51	4.25

The power conversion efficiency (*η*) and fill factor (FF) of the DSSCs were calculated using eqn (5) and (6):5
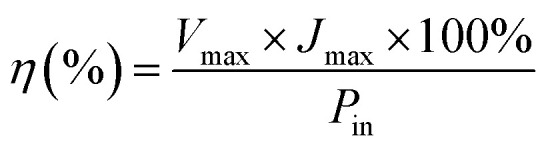
6
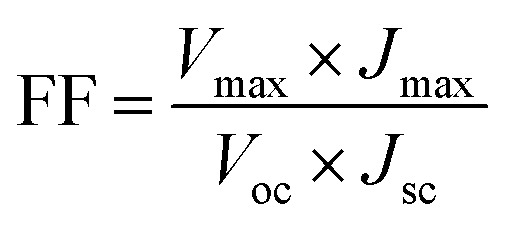
where *J*_max_ (mA cm^−2^) and *V*_max_ (V) are the current density and voltage at the point of maximum power output in the *J*–*V* curves, *P*_in_ is the light intensity of the incident light, *V*_oc_ is the open-circuit voltage (V) and *J*_sc_ is the short-circuit current density (mA cm^−2^) respectively.^86^

The DSSCs prepared using the carbon material show a short circuit current density *J*_sc_ of 11.00 mA cm^−2^, fill factor (FF) of 0.51, open circuit voltage *V*_oc_ of 0.75 V and conversion efficiency (*η*) of 4.25%. The DSSCs fabricated using platinum as the counter electrode showed a short circuit current density *J*_sc_ of 12.40 mA cm^−2^, fill factor (FF) of 0.68, open circuit voltage *V*_oc_ of 0.69 V and conversion efficiency (*η*) of 5.86% as demonstrated in Table 3. The carbon material exhibits a lower conversion efficiency than the Pt electrode although the carbon material exhibits higher redox reactivity. DSSCs fabricated from the carbon material also show a lower fill factor compared to the platinum-based cell while the open circuit voltage *V*_oc_ is higher compared to that of the platinum-based cell. The higher *V*_oc_ in carbon based DSSC is probably due to the low charge recombination rate compared to that of the Pt based DSSC while the low *J*_sc_ and fill factor may be due to the short electron lifetime and high internal resistance from ion diffusion. The low short-circuit current (*J*_sc_) and higher *V*_oc_ correlate with the charge of the carbon atom. This may be attributed to electrostatic interactions between the carbon atom and I^−^ or I_3_^−^, and a higher concentration of mediator anions in proximity to the carbon surface, resulting in the increase of the regeneration and recombination rates.^87,88^ The lower fill factor and *J*_sc_ are responsible for the poor efficiency. Even though a higher catalytic activity was observed in the electrode fabricated using the produced carbon due to the highly porous film offering a higher effective surface area for the reduction of triiodide species, the resistance of the film or higher overpotential towards the redox reaction appears to be the dominating factor in deciding the cell performance. The efficiency of carbon-based DSSCs can be improved by increasing the thickness of the counter electrodes because a higher catalytic activity may be observed in the carbon-based electrode due to the highly porous film. The porosity of the carbon film will increase with increasing thickness and it will offer a higher effective surface area for the reduction of tri-iodide species.^60^

## Conclusions

A carbon material was produced by carbonization of an organic ligand (Mq) at high temperature in flowing argon gas. The carbon material showed a graphitic nature and exhibited catalytic properties. The carbon material was utilized to prepare the counter electrode in DSSCs. The cell prepared with the carbon material exhibited an open circuit voltage *V*_oc_ of 0.75 V, short circuit current density *J*_sc_ of 11.00 mA cm^−2^, fill factor (FF) of 0.51 and conversion efficiency (*η*) of 4.25%. The DSSCs made using the carbon showed higher *V*_oc_ than Pt based DSSCs, and this may be due to the low recombination rate. The low *J*_sc_ and FF may be due to the higher overpotential towards the redox reaction or resistance of the carbon film. These factors seem to be the dominating factors in deciding cell performance. The efficiency of carbon based DSSCs can be enhanced with increasing the thickness of the counter electrodes.

## Conflicts of interest

There are no conflicts to declare.

## Supplementary Material
